# Monthly Summary

**Published:** 1898-06

**Authors:** 


### Monthly Summary.

### A NEW FUNCTION OF THE ANTRUM.

In the text-book we have been taught that the antrum has two functions, namely ; the lubrication of the nasal passages, and imparting warmth to the air as it passes into the lungs.

These so-called functions have seemed to us as extremely absurd. In the first place, the Schneiderian membrane is fully capable of supplying the necessary moisture to the nares; and in the second instance, the "crow-quill size" of the middle meatus is totally inadequate to afford much, if any, warmth to the large volume of air constantly passing its orifice. We have long entertained the belief that the antra were devoid of any function whatever, but in the divine plan were intended as an economizer of bone tissue; but above all, the better to minimize the consequence of fracture of the superior maxille.

A fall or blow on the outer wall of the antrum rarely involves its posterior aspect or immediate articulating bones, but were the maxilla solid bone structure, a fracture of those bones would be attended with more serious results than any that have yet been recorded.

In support of this proposition we direct attention to the well-grounded theory respecting the function of the sutures of the cranial bones.

Not to discuss the subject further by seeking analogies, we note a beautiful theory recently advanced by Dr. Sudduth, in which we heartily concur.

Speaking of the "Antrum and Vocal Resonance," he declares that the air contained in these cavities vibrates in harmony with the tones produced by the vocal cords. That this vibration is most appreciable when the tones produced are full of melody, as in certain kinds of church music and negro melodies. That it is more prominent in singing than in speaking, unless a special declamatory effect is attempted. That there is a type of individual to which the successful vocalist and orator belongs, and which is indicated, among other things, by a considerable but harmonious development of the maxillary sinuses. Their variation in the size and shape of the resonant cavities when present undoubtedly affects their value as resonators.—I. A. C., in *Dental Weekly*.

---

FLOSS SILK.—How often writers and speakers fail to mention floss silk as a means of dental prophylaxis. The tooth-brush is stressed, even to the number and shape of the rows of bristles; while the floss silk, which is far more important, is not mentioned, or if mentioned, only in a casual way.

Instructions at the chair are given in the use of the brush, which is well, but the silk should receive the same attention. Really, if one is to be neglected let it be the brush, which does not reach the seat of nine-tenths of the decayed places, however faithful and careful it is used. Impress on the patient the importance of keeping accumulation from between the teeth, where most caries are found. We have been for years using the Corticelli embroidery silk, found at dry goods stores on small spools of about two and one-half yards each, and which sell for one cent. We use this waxed, at the chair, and present a spool with instructions how to use, and to wax, to each new patient. So long and persistently have we urged this in our practice that patients say they feel uncomfortable if their teeth are not "cleaned between." Especially should patients for whom approximal fillings have been made be urged to keep them clean between. Tell them teeth cannot be made better than they were before they decayed, and if the same conditions are allowed to exist after they are filled that existed before, decay will surely recur around the fillings.

With many, if this is shown in the light of dollars and cents, it is more impressive. But with most intelligent people there is a desire to keep the teeth clean, and they only need to be given the proper directions for doing so, and will do it gladly.—ED. in *Dental Weekly*.

FILLING MATERIALS.—Dr. C. N. Johnson, Chicago.—The relative value of filling materials, it seems to me, relates more particularly to the individual requirements of each case than any other one thing. As an illustration, I had a patient to-day for whom I did some work, and I want to say a word or two about her, because it is an extreme illustration of the point I just made. It was a young girl, brought to me by her sister, who said that they had practically given up the idea of having her teeth attended to on account of the fact that she positively would not permit any dentist to work on her mouth She had been to several different dentists, and she was such an uncontrollable individual that no dentist would be bothered with her. I do not blame my fellow practitioners. The girl comes from a good family and ought to have some sense. But in all my experience I have never seen such a combination of pure cussedness in any patient as was concentrated in this girl. Her teeth were in bad condition. There was only one filling in the mouth, and that was an amalgam filling on the mesial surface of a lower molar and was not well inserted. But it was a marvel to me how it was inserted as well as it was. She was not ordinarily a nervous child, but the moment she took the chair she was apprehensive, not because she had been hurt previously, but had heard stories about the pain inflicted by dentists, and did not endeavor to have the least control of herself. She was not one of those individuals with whom one could reason. I have been ordinarily successful in managing children of a nervous temperament, but when I began to work on that child my first impulse was to dismiss her as the other dentists had done, and was on the verge of telling her to get out of the chair. Then the thought struck me that if I let her go, her teeth would be lost, for I felt sure they would not take her to any other dentist. So I resolved to put all my ingenuity into the case, and manipulated her as gently as I could, biting my lips much of the time to keep back the censure which I felt like giving her. At the final sitting to-day I have controlled her to the point of doing definite work on her teeth. Gold here has no place as a filling material. The most valuable filling material I know of in such a case is cement. Cement will stick where gutta-percha will not. I filled all of these teeth with cement. I have made an impression on that child and she will come back in the fall when I shall do better work for her.—*Dental Review.*

---

I WAS visiting a dentist a time ago when a well-dressed, intelligent looking young lady entered the office, saying :—

"Mrs. Judge Oliver recommended me to come to you to have some dental work done."

"Take off your wraps, if you please and take the chair," said he. "Yes, I see there is some work to be done. I will take a chart of it. What may I call your name ?"

"Mary Burgoin."

"Ah, O, yes. Mary, I believe you are working for Mrs. Oliver."

"Yes; I am her seamstress."

"Ah, yes—I am sorry; but Miss Burgoin, excuse me. I do not work for servants."

And he left her to leave the chair and put on her wraps and leave without any attendance.

I call that shabby, shameful, mean, ungentlemanly, confoundedly contemptible.—*Dental Brief.*

---

DANGER FROM AIR INJECTION.--Dr. McCormick.—As for ordinary hypodermic medication, or serum administration, we believe the danger from air injection is *nil.* In using the Koch syringe for giving small measured quantities of serum, toxins, etc., it is customary to be sure that the entire amount of fluid is injected, to allow one or more small bubbles of air to escape from the needle. During the past six years I have made thousands of such injections into rabbits, guinea-pigs, rats and mice, and have yet to see any harm.—*Jour. Amer. Med. Soc.*

---

A twenty-five per cent. solution of iodid of potassium removes blood stains.

